# *In vitro* and *in vivo* activity and cross resistance profiles of novel ruthenium (II) organometallic arene complexes in human ovarian cancer

**DOI:** 10.1038/sj.bjc.6600290

**Published:** 2002-05-03

**Authors:** R E Aird, J Cummings, A A Ritchie, M Muir, R E Morris, H Chen, P J Sadler, D I Jodrell

**Affiliations:** Cancer Research UK, Edinburgh Oncology Unit, Western General Hospital, Crewe Road South, Edinburgh EH4 2XR, UK; Department of Chemistry, University of Edinburgh, Edinburgh EH9 3JJ, UK

**Keywords:** ruthenium(II), organometallic arene complexes, structure activity relationships, drug resistance, ovarian xenografts

## Abstract

Ruthenium complexes offer the potential of reduced toxicity, a novel mechanism of action, non-cross resistance and a different spectrum of activity compared to platinum containing compounds. Thirteen novel ruthenium(II) organometallic arene complexes have been evaluated for activity (*in vitro* and *in vivo*) in models of human ovarian cancer, and cross-resistance profiles established in cisplatin and multi-drug-resistant variants. A broad range of IC_50_ values was obtained (0.5 to >100 μM) in A2780 parental cells with two compounds (RM175 and HC29) equipotent to carboplatin (6 μM), and the most active compound (HC11) equipotent to cisplatin (0.6 μM). Stable bi-dentate chelating ligands (ethylenediamine), a more hydrophobic arene ligand (tetrahydroanthracene) and a single ligand exchange centre (chloride) were associated with increased activity. None of the six active ruthenium(II) compounds were cross-resistant in the A2780cis cell line, demonstrated to be 10-fold resistant to cisplatin/carboplatin by a mechanism involving, at least in part, silencing of MLH1 protein expression via methylation. Varying degrees of cross-resistance were observed in the P-170 glycoprotein overexpressing multi-drug-resistant cell line 2780^AD^ that could be reversed by co-treatment with verapamil. *In vivo* activity was established with RM175 in the A2780 xenograft together with non-cross-resistance in the A2780cis xenograft and a lack of activity in the 2780^AD^ xenograft. High activity coupled to non cross-resistance in cisplatin resistant models merit further development of this novel group of anticancer compounds.

*British Journal of Cancer* (2002) **86**, 1652–1657. DOI: 10.1038/sj/bjc/6600290 www.bjcancer.com

© 2002 Cancer Research UK

## 

Metal complexes remain an important resource for the generation of chemical diversity in the search for novel therapeutic and diagnostic agents, especially in the arena of anticancer drug development (Guo and Sadler, 1999). *Cis*-dichlorodiammine platinum(II) (cisplatin) represents one of the most active and clinically useful agents used in the treatment of cancer, achieving cures in testicular cancer and high response rates in ovarian and small cell lung cancer (Giaccone, 2000). Evidence from both pre-clinical studies and clinical investigations has strongly implicated DNA as the biologic target for cisplatin through the formation of irreversible adducts via the process of ligand exchange (Jamieson and Lippard, 1999; Kelland, 2000). However, in common with many other cytotoxic drugs, cisplatin induces normal tissue toxicity, particularly to the kidney, and the development of acquired drug resistance can occur in initially responsive disease types (ovarian and small cell lung) or be present as intrinsic drug resistance in less-responsive disease types (non-small cell lung and colon) (Giaccone, 2000).

Based predominately on evidence from cell lines, the mechanistic basis for cisplatin resistance appears to be multi-factorial, involving drug transport defects (both reduced uptake and active efflux), detoxification by interaction with cellular nucleophiles, modulations in DNA repair pathways, and altered cellular signalling responses downstream of DNA damage (Fink *et al*, 1998; Johnson *et al*, 1998; Wang *et al*, 2000). A variety of approaches have been adopted to overcome both *de novo* and acquired cisplatin resistance, ranging from broad-based programmes of new platinum analogue design to specific attempts to modulate a single pathway of resistance (Judson and Kelland, 2000; Mimnaugh *et al*, 2000; Plumb *et al*, 2000).

The metal ruthenium (Ru) possesses several favourable chemical properties that indicate it may be a strong candidate to form a basis for rational anticancer drug design (Clarke *et al*, 1999; Allardyce and Dyson, 2001). Ru complexes demonstrate similar ligand exchange kinetics to those of platinum (Pt II) while displaying only low toxicity, which in part is believed to be due to the ability to mimic iron in binding to plasma proteins including transferrin and albumin (Allardyce and Dyson, 2001). Transport and sequestration of Ru into tumour cells may be mediated via protein transport and receptor mediated uptake (Guo and Sadler, 1999). Due to differing ligand geometry between their complexes, Ru compounds bind to DNA forming predominately inter-strand crosslinks as opposed to the intra-strand crosslinks favoured by cisplatin (Fruhauf and Zeller, 1991; Gallori *et al*, 2000). In addition, non-nuclear targets, such as the mitochondrion and the cell surface, have also been implicated in the antineoplastic activity of Ru complexes, particularly in the case of the clinically investigated Ru(III) antimetastatic drug (*trans*-RuCl_4_(DMSO)Im)(ImH) (NAMI-A) (Bergamo *et al*, 2000). Thus, Ru complexes offer the potential over Pt (II) complexes of reduced toxicity, a novel mechanism of action, the prospect of non-cross resistance (Zeller *et al*, 1991; Coluccia *et al*, 1993) and a different spectrum of activity. Previous investigators have focused on Ru(III) complexes as potential antitumour agents (Keppler *et al*, 1987; Berger *et al*, 1989; Seelig *et al*, 1992). In the present study, a series of novel Ru(II) organometallic arene complexes (Morris *et al*, 2001) have been evaluated for activity in both *in vitro* and *in vivo* models of human ovarian cancer, and cross-resistance profiles established in cisplatin and multi-drug resistant (MDR) variants.

## MATERIALS AND METHODS

### Investigational agents

**Figure 1:**
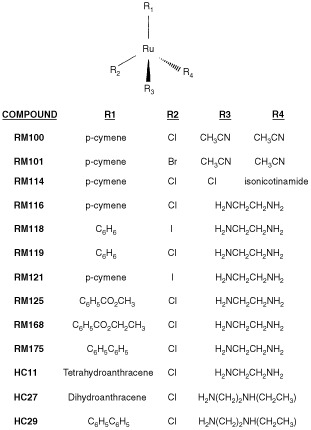
Molecular formulae of novel Ruthenium (II) organometallic complexes.

The 13 novel Ru(II) organometallic arene compounds (for formulae see Figure 1

) were synthesised and chemically characterised as described in detail (Morris *et al*, 2001) and are quoted in the following patents: PCT/GB00/04144 and PCT/GB01/02824. All compounds were salts with PF_6_^−^ as counter anion. Cisplatin, carboplatin and doxorubicin were obtained from the Western General Hospital pharmacy.

### Cell lines

The A2780 human ovarian cancer cell line and its MDR counterpart 2780^AD^ were kindly provided by Drs TC Hamilton and RF Ozols, Fox Chase Cancer Center, Philadelphia, USA. 2780^AD^ was cultured in the presence of 2 μM doxorubicin, which was removed 1 week prior to experimentation with the Ru(II) compounds. The A2780cis cell line was obtained from the ECACC (European collection of cell culture, Salisbury, UK). Cells were grown as monolayers in RPMI 1640 medium with L-glutamine (Gibco BRL, Paisley, UK) supplemented with 5% foetal calf serum and penicillin (110 U ml^−1^) and streptomycin (100 μg ml^−1^) and were maintained under standard tissue culture conditions of 37°C and 5% CO_2_. Experiments were performed on cells within 10 passages of each other.

### *In vitro* growth inhibition assays

The human ovarian cells were added at a density of 1×10^4^ cells per well to 24-well tissue culture trays (Falcon Plastics, Becton Dickinson, Lincoln Park, NJ, USA) and allowed to grow for 72 h before addition of the Ru(II) arene complexes. Stock solutions of the ruthenium compounds were made up fresh in deionised water and sonicated to ensure complete dissolution. These stock solutions were diluted with media to give final concentrations ranging between 0.1 and 100 μM. All the compounds were evaluated at each concentration in duplicate wells, and complete assays were repeated a minimum of three times. Cisplatin or carboplatin was employed as a positive and comparative control in each experiment. After 24-h exposure the drug-containing medium was removed, the cells washed with phosphate buffered saline (PBS) and fresh medium was added. Cell number was assessed after a further 72 h growth using a Coulter counter (Coulter Electronics Ltd, Luton, UK) and the IC_50_ values (concentration of drug causing 50% growth inhibition) calculated by linear regression analysis comparing the inhibitory effects of the drugs against the growth of untreated cells.

### Co-incubation of human ovarian cell lines with verapamil or 2′deoxy-5-azacytidine (DAC)

Cells were cultured in 24-well plates as above. Verapamil (50 μM, Sigma Chemical Co., Poole, UK) was added concomitantly with the Ru(II) test compounds or Pt(II) compounds and doxorubicin control for 24 h and growth inhibition was then established as above. DAC (0.5 μM, Sigma) was added to cells for 3 days prior to being removed with PBS washes and then the cells were treated with cisplatin for 24 h and growth inhibition was then established as above.

### Immunoblotting for MLH1

A2780, A2780cis and 2780^AD^ cells were plated down at a density of 5×10^5^ cells per well in 6-well trays and harvested when 70% confluent. Extracts were prepared by suspending the cell pellets in 50 mM HEPES (pH7.4), 1% Triton X-100, 150 mM NaCl, 5 mM EDTA and 0.5% sodium deoxycholate in the presence of a standard cocktail of protease inhibitors (Sigma). Protein concentrations were determined by the Bio-Rad protein assay (Bio-Rad, Richmond, CA, USA). Samples (20 μg) were denatured and separated on a 10% SDS polyacrylamide gel before being transferred to nitrocellulose and probed using a mouse monoclonal antibody for hMLH1 (Clone G168-15, Pharmingen). Blots were visualised by ECL detection (Santa Cruz). Levels of protein loading were examined using an actin antibody (Calbiochem). In a separate study, A2780 and A2780cis cells were treated with 10 μM DAC for 3 days prior to protein extraction and SDS polyacrylamide gel electrophoresis as above.

### Antitumour activity in human ovarian cancer xenografts

The antitumour activity of the novel Ru(II) organometallic arene complexes was evaluated in A2780, 2780^AD^ and A2780cis xenografts growing in *nu/nu* mice essentially as described previously (Cummings *et al*, 1996). The three xenografts were established from their respective cell lines by subcutaneous implantation of 5×10^6^ cells in serum free media in the flank of animals. The resultant xenografts were subjected to regular pathologic examination to confirm the human ovarian cancer phenotype. All animal experiments were carried out with local and The Imperial Cancer Research Fund ethical committee approval. The ethical guidelines that were followed meet the standards required by the UKCCCR guidelines (Workman *et al*, 1998). Female *nu/nu* mice were implanted with 2–3 mm^3^ fragments of viable tumour and left for approximately 3–4 weeks. Animals were randomised into control and drug treated groups, each of which contained 5–10 mice. Ru (II) complexes were administered as 10% DMSO solutions in sterile saline and cisplatin as a solution in sterile saline both at a volume of 0.1 ml/10 g of body weight i.p. Tumour volumes were determined by calliper measurement and calculated using the formula: volume=0.5×length×width^2^. Relative tumour volume (RTV) was then calculated for each individual tumour by dividing tumour volume on day t by the tumour volume on day 1×100%. T/C (%) values were calculated as the mean tumour volume of the drug treated group/mean tumour volume of the control group×100 from the data generated as indicated below. Antitumour activity studies were performed on either two or three separate occasions.

## RESULTS

*Structure activity relationship for Ru(II) organometallic arene complexes in A2780 human ovarian cancer cells*

The 13 organometallic complexes were initially screened against the platinum sensitive human ovarian cancer cell line A2780 in order to establish a baseline level of growth inhibitory activity (Brown *et al*, 1993). A broad range of IC_50_ values was obtained (see Table 1

**Table 1:**
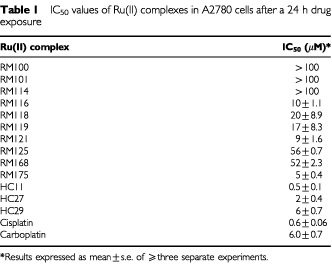
IC_50_ values of Ru(II) complexes in A2780 cells after a 24 h drug exposure

) with two compounds (RM175 and HC29) equipotent to carboplatin (6 μM), one compound (HC11) equipotent to cisplatin (0.6 μM) and one compound (HC27) intermediate in potency between the two platinum complexes. Three compounds (RM 100, 101 and 114) recorded values greater than 100 μM and were defined essentially as inactive, and the remaining six compounds were of intermediate value (IC_50_, 9–56 μM). Of interest, RM100, RM101 and RM114 when originally tested shortly after synthesis yielded IC_50_ values of 7, 8 and 11 μM respectively, but these decayed over a period of 12 weeks to >100 μM even while stored in the solid state at 4°C. These compounds alone contained more reactive monodentate ligands such as acetonitrile at positions R3 and R4 (see Figure 1) in comparison to the more stable chelating ligand ethylenediamine (en) or N-ethyl ethylenediamine employed in the remaining compounds. Substitution of the proposed halide reactive centre (R2, Figure 1) with iodide as opposed to chloride had little effect on *in vitro* potency (compare RM118 with RM119 and RM116 with RM121). Minor chemical alterations to the en chelating ligand (HC29 *vs* RM175, Figure 1 and Table 2

**Table 2:**
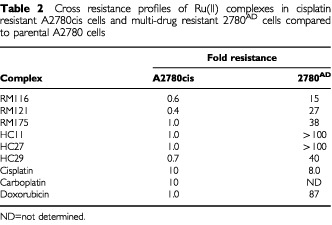
Cross resistance profiles of Ru(II) complexes in cisplatin resistant A2780cis cells and multi-drug resistant 2780^AD^ cells compared to parental A2780 cells

) also had little effect on potency. However, substitution at position R1 (the arene ligand) from benzene (RM119) to p-cymene (RM116) to biphenyl (RM175) to dihydroanthracene (HC27) to tetrahydroanthracene (HC11) increasing overall hydrophobicity resulted in a large increase in growth inhibitory activity from (IC_50_) 17 to 10 to 5 to 2 to 0.5 μM. The arene ligand is believed to play a role in preventing oxidation of the ruthenium from oxidation state II (the proposed biologically active form) to oxidation state III (proposed inactive, prodrug form) (Allardyce and Dyson, 2001; Morris *et al*, 2001). It may also facilitate cellular uptake of the compounds.

### Cross resistance profiles of Ru(II) organometallic arene complexes

The fold resistance values for the six most active compounds RM116, 121, 175 and HC11, 27 and 29 in the 2780^AD^ MDR and A2780cis cisplatin resistant variants are listed in Table 2. Compared to the parental cell line (A2780), a relatively high degree of cross-resistance was observed in 2780^AD^ ranging from a factor of 15 for RM116 up to >100 for HC11 and this is compared to 87-fold for doxorubicin, the original selection agent (Louie *et al*, 1986) (see Table 2). In addition, there was an eight-fold level of resistance to cisplatin in 2780^AD^. By contrast, in A2780cis cells the six organometallic complexes together with doxorubicin were completely non-cross resistant, while cisplatin and carboplatin were both 10-fold cross resistant (Table 2).

### Mechanisms of drug resistance

**Figure 2:**
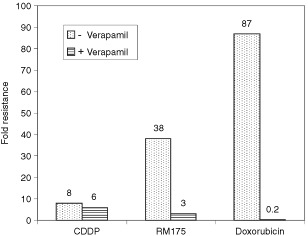
Effect of co-administration of 50 μM-verapamil on the degree of cross-resistance exhibited in the 2780^AD^ multi-drug resistant cell line to doxorubicin, cisplatin and the novel Ru(II) arene complex RM175 (for structure see Figure 1).

Verapamil has been shown to effectively abrogate P-gp mediated active efflux of anticancer drugs in ovarian cancer cells by competitive inhibition of drug transport and reverse multi-drug resistance (Rogan *et al*, 1984). The influence of verapamil on the level of cross-resistance in 2780^AD^ to RM175, cisplatin and doxorubicin was evaluated. With doxorubicin, 50 μM verapamil restored the chemosensitivity of the MDR cell line back to that of the parental cell line (Figure 2

). In a previous study we have demonstrated that verapamil was able to completely abolish doxorubicin active efflux in 2780^AD^ (Cummings *et al*, 1996). As anticipated, verapamil had little effect on the activity of cisplatin, consistent with its known lack of recognition by P-gp (Allen *et al*, 2000). In the case of the Ru(II) compound RM175, verapamil was also highly effective at restoring sensitivity (Figure 2). Here, cross resistance fell from a factor of 38 to only three-fold.

**Figure 3:**
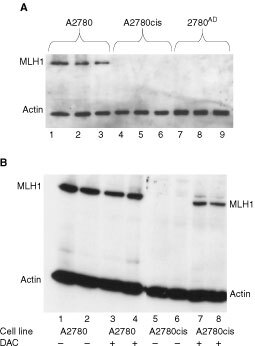
(**A**) MLH1 protein levels in the A2780 (lanes 1–3), 2780cis (lanes 4–6) and 2780^AD^ (lanes 7–9) determined by Western blot analysis. (**B**) MLH1 protein levels in the A2780 and A2780cis cell lines plus (+) or minus (−) co-incubation with 10 μM 2′deoxy-5-azacytidine (DAC) for 3 days prior to protein extraction and SDS polyacrylamide gel electrophoresis and Western blotting.

Cisplatin resistance is frequently associated with loss of proficiency of the mismatch repair pathway due to methylation and silencing of the MLH1 gene (Brown *et al*, 1997; Plumb *et al*, 2000). The A2780cis cell line was confirmed to lack MLH1 protein as well as 2780^AD^, while the parental cell line expressed the protein (Figure 3a

). Treatment with the demethylating agent DAC had little effect on MLH1 protein levels in parental A2780 cells (Figure 3b). However, it recovered expression of the protein in the A2780cis cell lines almost back to that of the wild-type cells (Figure 3b). In addition, DAC was able to partially reverse drug resistance to cisplatin in A2780cis cells by a factor of two-fold.

### Antitumour activity of RM175

**Figure 4:**
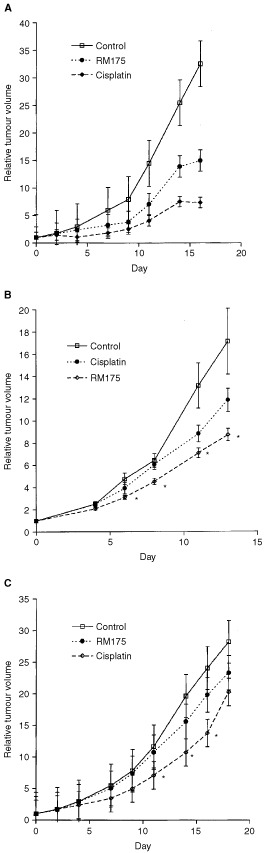
Antitumour activity of RM175 administered on days 1 and 5 at 25 mg kg^−1^ i.p. *vs* cisplatin administered on day 1 at 10 mg kg^−1^ i.p. (**A**) Effect of RM175 and cisplatin on the A2780 human ovarian cancer xenograft. (**B**) Effect of RM175 and cisplatin on the A2780cis human ovarian cancer xenograft and (**C**) effect of RM175 and cisplatin on the 2780^AD^ human ovarian cancer xenograft. Statistical significance was evaluated by Student's *t*-test comparing the drug treated groups at specific time points against the control group: *P<0.05. In the case of A2780, cisplatin was more active than RM175 on days 14 and 16 (*P*<0.01), while in A2780cis RM175 was more active than cisplatin on days 8 and 13 (*P*<0.05).

In preliminary studies it was established that the maximum tolerated dose (MTD) of cisplatin was 10 mg kg^−1^ i.p. as a single administration, in keeping with previous reports (Langdon *et al*, 1994). Mice were able to tolerate up to 25 mg kg^−1^ RM175 on days 1 and 5 without significant weight loss. At the above dose regimen, RM175 produced a significant growth delay against the A2780 xenograft representing a T/C value of 46% on day 16 (Student's *t*-test compared to controls, Figure 4a

). By comparison cisplatin at its MTD produced a T/C value of 23% on day 16 of the study (Figure 4a). In the A2780cis xenograft RM175 growth inhibitory activity was maintained (T/C 51%, day 13) and was significantly greater than that of cisplatin on day 8 (*P*=0.03) and day 13 (*P*=0.01) (Figure 4b). Cisplatin was active in the 2780^AD^ xenograft, although not to the same level as the parental xenograft (T/C value 55 %, day 14), while RM175 was inactive against 2780^AD^ (Figure 4c). Thus, the patterns of activity established *in vitro* for both RM175 and cisplatin were mirrored to a large degree *in vivo*, making the A2780 cell line and its two resistant clones an attractive pre-clinical screen for evaluating new agents in this class (Workman *et al*, 1998).

## DISCUSSION

The aim of the present study has been to evaluate the *in vitro* and *in vivo* the activity of a novel series of Ru(II) organometallic arene complexes and identify potential resistance mechanisms, with the intention of feeding back this information into rational design of second generation compounds. Ru(II) arene complexes with *in vitro* potency greater than that of carboplatin and equal to cisplatin were identified, and *in vivo* xenograft activity was established in human ovarian cancer. A pattern of cross-resistance emerged for the Ru(II) arene compounds, quite distinct from that of cisplatin and carboplatin, characterised by non-cross resistance in cisplatin resistant cells (and xenograft) but cross-resistance in multi-drug resistant (MDR) cells (and xenograft).

The 2780^AD^ cell line utilised in the present study displays the classic MDR phenotype mediated via over-expression of the 170 kD plasma membrane glycoprotein P-gp and reduced cellular drug accumulation (Cummings *et al*, 1995, 1996). In addition, multi-drug (associated) protein 2 (MRP2) is up-regulated in this cell line (Cummings *et al*, unpublished observations). Over-expression of MRP2 is a common phenomenon in human ovarian cell lines (including A2780) that display higher levels of resistance to cisplatin (Borst *et al*, 2000; Kool *et al*, 1997). However, MLH1 protein was also shown to be absent, which may be responsible for the eight-fold level of drug resistance observed with cisplatin in 2780^AD^ (Table 2). P-pg displays striking substrate specificity for naturally occurring hydrophobic molecules, particularly those carrying a positive charge but does not transport cisplatin (Endicott and Ling, 1989; Gottesman and Pastan, 1993; Seelig *et al*, 2000). By contrast, MRPs 1, 2 are 3 are well recognised as transporting organic anions, or neutral compounds as either glutathione (GSH) conjugates or co-transported with GSH (Borst *et al*, 2000; Ishikawa *et al*, 2000; Zeng *et al*, 2000). The six organometallic complexes investigated for cross-resistance in the present study (see Table 2 and Figure 1) exhibit both of the features – hydrophobicity and a cationic centre – that facilitate substrate recognition by P-pg (Endicott and Ling, 1989; Gottesman and Pastan, 1993; Seelig *et al*, 2000). Thus, it is likely that the cross-resistance to Ru(II) arene complexes observed in the 2780^AD^ cell line is due, at least in part, to recognition and active efflux by P-pg. The almost complete reversal of RM175 drug resistance by verapamil suggests that P-gp may function as the pre-dominant or even sole drug-resistance mechanism in this cell line. It is noteworthy that the more hydrophobic the organometallic complex, the higher the level of intrinsic drug potency in A2780 parental cells, but also the greater the degree of cross-resistance in 2780^AD^.

The mechanism of resistance to cisplatin in A2780cis cells remains poorly characterised (ECACC catalogue, European collection of cell culture, Salisbury, UK). A number of potential resistance mechanisms to cisplatin and analogous alkylating agents in a variety of A2780 clones have been reported previously including:

- loss of proficiency of the mismatch repair pathway (MMR) due to methylation and silencing of the *hMLH1* gene (Brown *et al*, 1997) (Plumb *et al*, 2000)
- accumulation of the mutant (inactive) form of the p53 tumour suppressor gene (Brown *et al*, 1993)
- elevated GSH cellular content and a concomitant increase in expression of MRP2 (Kool *et al*, 1997) and
- altered DNA damage recognition, cellular signalling and DNA repair (Roy *et al*, 2000)

Since the novel Ru(II) organometallic complexes were unaffected by these putative mechanisms, it was important to establish the mechanism(s) of cisplatin resistance in A2780cis. In the present study, Western blot analysis of the MLH1 protein in A2780 parental (cisplatin sensitive) cells and A2780cis (cisplatin resistant) cells clearly demonstrated that it was not present in the resistant cell line. Treatment of A2780cis cells with DAC re-established MLH1 protein expression and partially reversed drug resistance to cisplatin. DAC has recently been shown to reverse cisplatin drug resistance in the related A2780/cp70 xenograft by demethylation of the *hMLH1* gene promoter (Plumb *et al*, 2000). Thus, it would appear that a deficiency in MMR is at least partially responsible for the expression of cisplatin resistance in the A2780cis cell line and by implication that MMR proficiency is not a prerequisite in the mechanism of action of the novel Ru(II) compounds, consistent with a number of other ruthenium complexes (Fruhauf and Zeller, 1991; Zeller *et al*, 1991; Coluccia *et al*, 1993; Kelland, 1999). MMR deficiency usually accounts for a 2–3-fold level of resistance (Fink *et al*, 1998) and in the present study co-administration of DAC was only able to reverse cisplatin resistance in A2780cis by two-fold indicating the possibility of the presence of other resistance mechanisms in this cell line. At present, studies are in progress in the A2780cis cell line to investigate the role of drug transport, expression of drug transporters, levels of GSH and the role of p53.

In summary, a series of novel Ru(II) organometallic arene complexes have been evaluated for activity both *in vitro* and *in vivo* in human ovarian cancer. Preliminary structure activity data have been obtained suggesting that stable (chelating) bystander ligands, a more hydrophobic arene ligand (possibly to enhance cellular penetration) and a single ligand exchange centre are associated with reproducible and increased growth inhibitory activity. These properties, particularly the hydrophobic arene group may also promote recognition by P-gp. Reduced *in vivo* toxicity and non cross-resistance in cisplatin resistant models *in vitro* and *in vivo* have been confirmed, justifying further development of this novel and interesting group of metal complexes.
